# Weak effects on growth and cannibalism under fluctuating temperatures in damselfly larvae

**DOI:** 10.1038/s41598-022-17192-1

**Published:** 2022-07-28

**Authors:** Kim Lea Holzmann, Chloé Charrier, Frank Johansson

**Affiliations:** Department of Ecology and Genetics, Uppsala University, 75236 Uppsala, Sweden

**Keywords:** Ecology, Zoology, Climate sciences

## Abstract

The Earth’s climate is changing with a trend towards higher mean temperatures and increased temperature fluctuations. Little attention has been paid to the effects of thermal variation on competition within species. Understanding the temperature-dependence of competition is important since it might affect dynamics within and between populations. In a laboratory experiment we investigated the effects of thermal variation on growth and cannibalism in larvae of a damselfly. The temperature treatments included three amplitudes between 20 and 26 °C with an average of 23 °C, and a constant control at 23 °C. Larvae were also raised at five constant temperatures for an estimation of the thermal performance curve, which showed that the thermal optimum for growth was 26.9 °C. Cannibalism was significantly positively correlated with initial body size variance. There was neither a difference among the temperature variation treatments, nor between the constant and the variation treatments in growth and cannibalism. Hence, positive and negative effects of temperature variation within the linear range of a species thermal performance curve might cancel each other out. Since our study mimicked natural temperature conditions, we suggest that the increase in temperature variation predicted by climate models will not necessarily differ from the effects without an increase in variation.

## Introduction

Climate change is mostly associated with increasing global temperatures, but larger temperature fluctuations are also predicted^1^. Such temperature fluctuations take place across multiple time scales^2^: long-term variations ranging from climate change to seasonal changes^3^, and short-term variations related to weather patterns on the scale of days to weeks^4^. A key question in ecology is how these short-term temperature variations, in the context of a progressively fluctuating climate, affect biotic interactions and the growth, development, and survival of organisms.

Insects comprise the majority of described animal species and can be extremely sensitive to climate change^5–7^. Several studies have found range shifts in insects and some of these changes are presumably due to climate changes^8–11^. However, we lack a detailed mechanistic understanding on what is causing this pattern. As insects are ectotherms, their basic physiological functions, such as locomotion and growth, highly depend on environmental temperatures^5,12^, and thus more information on how these variables are affected by temperature might help explaining and predicting range shifts.

Thermal performance of insects is usually estimated by measuring fitness-related traits over a range of temperatures^13^. Depending on the thermal performance properties of an individual and the mean temperature, thermal variation can either enhance or reduce performance^1^. In ectotherms, thermal performance curves (TPCs) are typically nonlinear and asymmetric, with three important components: (1) an initial increase in performance, (2) which flattens out and reaches a peak, and (3) a decrease in performance thereafter, which reduces at a faster rate than the initial increase. Therefore, temperature shifts result in uneven effects on performance depending on the position along the TPC^12^. This dual effect is usually referred to as Jensen’s inequality, a mathematical term for patterns that are independent of average conditions^14,15^. Most climate-change studies have focused on the effects of changing mean temperatures^16–20^, but the effects of thermal variation on organisms remain difficult to predict^21^.

Previous studies have shown contrasting effects of temperature variation on growth. In insects, growth was reported to be faster under fluctuating thermal conditions in studies from the early 1900s^22,23^. However, more recent studies have shown the opposite or no effect of temperature variation on growth in a variety of ectothermic taxa, such as insects, phytoplankton, and fish^24–27^. While most studies acknowledged that the effect of variation seems highly dependent on the experimental temperature range and the organism’s individual thermal performance parameters, few have included the actual TPC for accurate interpretation. This is unfortunate, because the predicted temperature response depends very much on where on the curve the temperature range fluctuations are occurring^28^. If for example, most of the temperature fluctuations are occurring on a part of the slope where there is a small change in slope along the curve, we would expect small differences between fluctuation temperatures and a constant temperature with the same mean temperature. This would also suggest the predicted increase of temperature fluctuations due to climate change would have a similar effect as constant increasing temperatures.

The effects of thermal variation on intraspecific interactions are highly understudied as previous research in the field of climatic variation focused mainly on the physiology of individuals^7,29^ but see^30–33^. Understanding the temperature-dependence of interactions within a population is important, as these interactions are strongly linked to an individual’s life history, involving reproduction and competition^34^. Interacting individuals can be affected by fluctuating temperatures because metabolic processes increase at warmer temperatures, resulting in faster movement and higher activity^35,36^. This can lead to more frequent encounters between individuals and consequently, to changes in the dynamics within populations and their biotic communities. In many taxa, such interactions between individuals can result in cannibalism, defined as intraspecific predation, or the killing and consumption of conspecifics^37^. Cannibalism is assumed to be directly beneficial for gaining additional nutrients, and indirectly through the removal of potential competitors^38^, but it also comes with costs: in carrying out attacks on conspecifics as well as defending against counterattacks^39^. Cannibalism has been shown to be temperature-dependent in previous studies^40–42^, but no studies on how temperature variation affects cannibalism exist.

A suitable model taxon for the investigation of impacts of climate change on growth and cannibalism are the Odonata, dragonflies and damselflies, which are temperature sensitive^43,44^, and important predators shaping community dynamics in freshwater systems^45,46^. The majority of development and growth takes place during the larval stage and is therefore important for adult fitness^47^, which in turn is impacted by an adult individual’s body size^48^.

In order to understand the consequences of anthropogenically induced climate change, it is important to include temperature variations, since fluctuations are expected to increase in the future^49–51^. For example, global climate models have shown that heat waves with a length of about a week are becoming more intense in the future^51^. We studied growth and cannibalism in the damselfly *Enallagma cyathigerum* (Charpentier, 1840) at constant and weekly fluctuating temperatures, which mirror realistic current environmental conditions, and weather patterns based on water temperatures from model-based predictions. In addition, to provide a mechanistic understanding of growth and cannibalism, we estimated the thermal performance curve of growth in *E. cyathigerum* using five different constant temperatures. Based on estimates of thermal performance parameters for *E. cyathigerum* (see^52^), and on simulated temperatures in the laboratory experiment—based on estimates of natural temperatures at our study site—we expected our experimental temperature range to be to the left but close to the species thermal optimum on the TPC. Thus, the change in performance during warm periods (closer to the peak of the TPC, i.e., flatter slope) would be less than the change in performance during the cold period (increasing part of the TPC, i.e., steeper slope), resulting in an overall lower mean performance compared to a constant mean temperature. We thus expected to find decreased growth and cannibalism with larger temperature variations compared to a constant temperature, due to Jensen’s inequality effects. In the discussion we provide potential suggestion how our results might affect future population distribution and dynamics.

## Methods

### Study system

Growth and cannibalism were studied in larvae of *Enallagma cyathigerum*, a species with a distribution throughout Eastern Palearctic and native to Sweden^53^. In Europe the distribution covers a south north gradient from southern Spain to northern Sweden^53^. Eggs were retrieved from wild females that were caught mated at a pond in the city of Uppsala, Sweden, 21 June 2021 (59.839142 N, 17.730666 E). After catching, females were placed in plastic cups (250 ml) lined with wet filter paper. The females oviposited their eggs into the filter paper after 2–3 days. Thereafter, the filter paper was placed in small one-liter plastic boxes filled with conditioned tap water holding a temperature of 20 °C. Larvae hatched between 7–8 July. After hatching, larvae were introduced into larger plastic containers (25 cm diameter, 12 cm height, filled with conditioned tap water, 20 °C) and kept at a photoperiod of 16:8 (light:dark period). After four weeks, winter conditions were simulated by placing the containers with the larvae in darkness at a temperature of 12 °C for two weeks. To simulate end of winter conditions, the containers with the larvae were moved back to the initial temperature and photoperiod. By first rearing the larvae under late summer photoperiod and temperature, and thereafter simulating winter and spring period close to natural photoperiod and temperature, cues were received. Such conditions are important for correct estimates of growth rate^54^. The pre-experimental rearing was conducted in walk-in climate rooms and the larvae were fed daily ad libitum with laboratory reared brine shrimps (*Artemia*). This food resource has been used successfully in previous experiments on damselflies^55,56^. Larvae were transferred into new containers for three experiments. These containers also included two grass stems of approximately 5 cm length per individual to simulate semi-natural vegetation structure and provide protozoans and bacteria. In experiment one, we estimated the thermal performance curve by comparing growth rates of individuals under five different constant temperatures. In experiment two, we estimated growth rates of individuals in the absence of potential competitors, referred to as the “single experiment”. In experiment three, we investigated growth and cannibalism among competing individuals, hereafter referred to as the “interaction experiment”. Experiment two and three were performed under both a constant and fluctuating temperatures where the average temperature during fluctuation was the same as in the constant treatment.

### Thermal performance estimates

To estimate a thermal performance curve on growth, 20 single *E. cyathigerum* larvae were reared at five constant temperatures of 17, 19, 22, 25 and 28 °C over nine weeks. At this time the larvae were in instar F4–F5. They were kept individually in plastic cups (7 cm diameter, 4.1 or 7 cm height) with tap water, which were placed in a water bath containing a heater (EHEIM thermocontrol, Deizisau, Germany) and circulator (AQUAEL, Suwalki, Poland). Larvae were fed daily (see study system). Growth was documented by taking images of the larvae in a petri dish, at the start and end of the experiment with a camera (Lumix, Panasonic Corporation, Japan, with a Leica F 2.8 lens) mounted on a tripod. Body size was estimated as head width (distance between the outer part of the eyes in dorsal view) from the photos taken using the software ImageJ version 1.53 (https://imagej.nih.gov/ij/)^57^. A graph paper (unit = 1 mm) under the petri dish served as a reference. Measurements of body size were repeated for a subset of 30 larvae across different replicates and treatments, and linear regression was used to confirm the high accuracy of measurements (r^2^ = 0.9984). The use of head width has been shown to be a good estimate of overall body size in damselflies^58^.

### Single experiment

To estimate growth rate in the absence of competitors, a treatment with single *E. cyathigerum* larvae was used to investigate individual growth rates at a constant temperature of 23 °C and at a high temperature variation (20–26 °C). We chose these temperatures because they are within the range of natural water temperatures at the study site and cover the predicted increase at about 2–3 °C until year 2080^59,60^. Forty individuals (20 per treatment) were kept singly in a plastic cup (7 cm diameter, 7 cm height) filled with water and fed daily (see study system). These cups were placed inside two water baths filled with tap water, an aquarium heater and a water circulator. The control group was exposed to a constant temperature of 23 °C and the other treatment to a continuously fluctuating temperature between 20 and 26 °C (Supplementary Fig. S1). A full cycle of thermal variation took two weeks, similar to natural weather patterns^4^, and a water temperature of 23 °C is within the range of the mean and maximum temperature at the latitude where the eggs were collected, see below under natural pond water temperature. The aquarium heaters were set to the minimum or maximum temperature (i.e., 20 or 26 °C) of the respective cycle each Monday and it took approximately 12 h until the water temperature reached these temperatures. This experiment underwent three full temperature variation cycles, which corresponds to a duration of six weeks. Each cup received an amount of 109 ± 13 (confidence interval = 0.95, n = 54) *Artemia*. Body size was measured as in the thermal performance estimates.

### Interaction experiment

Survival and growth of larvae reared at intraspecific competition under four different temperature settings were analyzed. Plastic containers (11 × 11 cm) holding 10 larvae (10 replicates) and filled with 450 ml of tap water, reflecting natural larval densities^61^, were placed in four separate water baths with heaters and water circulators. The control groups were exposed to a constant temperature of 23 °C, whereas the treatment groups varied on three weekly fluctuating temperature amplitude levels: low with 22–24 °C, medium with 21–25 °C and high with 20–26 °C (see Single Experiment and Supplementary Fig. S1). Four full temperature variation cycles, corresponding to eight weeks, were performed for all groups. Larvae were fed daily (see study system) and each container received an amount of 109 ± 13 (s.e.m., confidence interval = 0.95, n = 54) *Artemia* per larva. The location of larvae containers within the water baths was changed randomly every week after counting of larvae and the four chambers itself were rotated every second week to avoid possible room effects. Larvae were counted weekly each day after changing the temperature to record missing larvae as proxy for cannibalism. Non-cannibalism-related mortality, caused by for example developmental errors, will be referred to as “intrinsic mortality” and was estimated as carcasses. Body size was measured as in the thermal performance estimates.

### Natural pond water temperature

To estimate natural temperature in the pond where the *E. cyathigerum* larvae were collected, we used the online application FLake-Global (http://www.flake.igb-berlin.de/)^62^. This model is able to provide temperatures in natural freshwater based on meteorological data at the site of the water. To obtain realistic temperature settings for our experiment we used the simulation type “most recent lake response” available in the model, which is based on climate data from the year 2018 and allowed us to estimate recent environmental parameters of our collection site. Input parameters were lake mean depth, estimated as 1 m with intermediate turbidity (water transparency 1 m), and lake fetch (i.e., the maximum distance of open water the wind can travel) was calculated to be 100 m referring to the satellite image. Predicted increases of annual mean temperatures for the study region were investigated by running climate change scenarios for Uppsala county using SMHI, the Swedish Meteorological and Hydrological Institute (https://www.smhi.se/)^63^.

### Statistical analyses

All statistical analyses were conducted in R 4.1.2^64^ and results were considered significant at *α* = 0.05. Intrinsic mortality was calculated based on carcasses without sign of wounds found during the experiment and excluded for calculating cannibalism rates. Differences in intrinsic mortality across amplitude treatments and control were statistically tested with a one-way fixed-effects analysis of variance (ANOVA). A proxy for cannibalism was the number of larvae missing at the end of the experiment in the constant and the temperature variation treatment. Differences in cannibalism across treatments were tested with a binomial generalized linear model applying the glm function, and p-values were calculated using a chi-square test for the two categorical variables (survival and temperature treatment). Correlation between cannibalism and body size variance were tested with logistic regression using the lm function. Cannibalism data was log-transformed, and size variance data was squareroot-transformed to meet assumptions of a normal distribution. Growth rate in the interaction, single and thermal performance experiments was calculated as

1 $$growth = \frac{{\log \left( {final\,body\,size\,in\,mm) {-} {\text{log}}(initial\,body\,size\,in\,mm} \right)}}{time\,in\,days}.$$

In the interaction experiment, it was not possible to track individual larva, but all larvae alive were measured at the start and at the end of the experiment, and average values for each group of 10 larvae were used for calculation of growth (1). One-way ANOVAs were used to test for differences in initial and final body sizes as well as for growth rate differences across temperature variations. Thermal performance curves were estimated using the O’Neill function

2 $$rate = R_{max} \left( {\frac{{CT_{max} - temp}}{{CT_{max} - T_{opt} }}} \right)^{x} *exp\left( {x\left( {\frac{{temp - T_{opt} }}{{CT_{max} - T_{opt} }}} \right)} \right),$$

with $$x = \left( {\frac{{w^{2} }}{400}} \right)*\left( {1 + \sqrt {\left( {1 + \left( \frac{40}{w} \right)} \right)^{2} } } \right)$$ and $$w = \left( {Q_{10} - 1} \right)*\left( {CT_{max} - T_{opt} } \right)$$.

This function (2) has previously been used successfully for Odonata, see Suhling et al.^65^. The function takes the rise of growth rate with increasing temperatures (temp) into account and drops again after reaching a peak, the typical shape of TPCs^12^. Critical maximum (CT_max_) and optimal (T_opt_) temperatures, as well as maximum performance rate at optimum temperature (R_max_) and Q_10_, which defines the fold change in performance as a result of increasing the temperature by 10 °C, were estimated in R using the rTPC package with a nonlinear least square approach based on the “oneill_1972” model^66^.

## Results

### Thermal performance estimates

**Figure 1:**
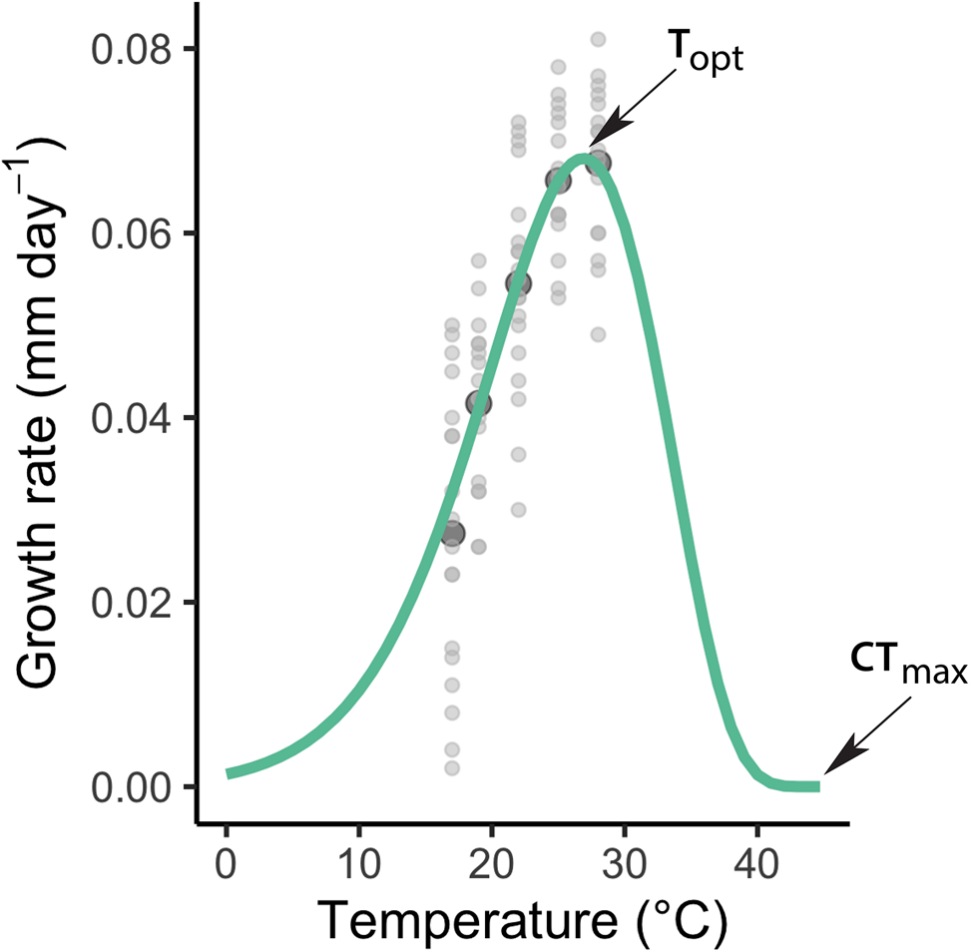
Thermal performance curve (TPC) with growth rate (mm day^−1^) in relation to the temperature (°C) for *Enallagma cyathigerum* calculated with the O’Neill function. Light grey dots are the observed growth rates and dark grey indicate the mean values. CT_max_, critical maximum temperature; T_opt_, optimum temperature.

Growth was estimated to be the highest at a temperature of 26.9 °C (T_opt_) with a maximal growth rate of 0.0681 mm day^−1^ (R_max_), and a Q_10_ value of 2.56 (Fig. 1). The upper critical thermal limit (CT_max_) was estimated as 44.7 °C (Fig. 1). The estimated T_opt_ is within the range observed empirically for other Coenagrionid species^65^.

### Single experiment

**Figure 2:**
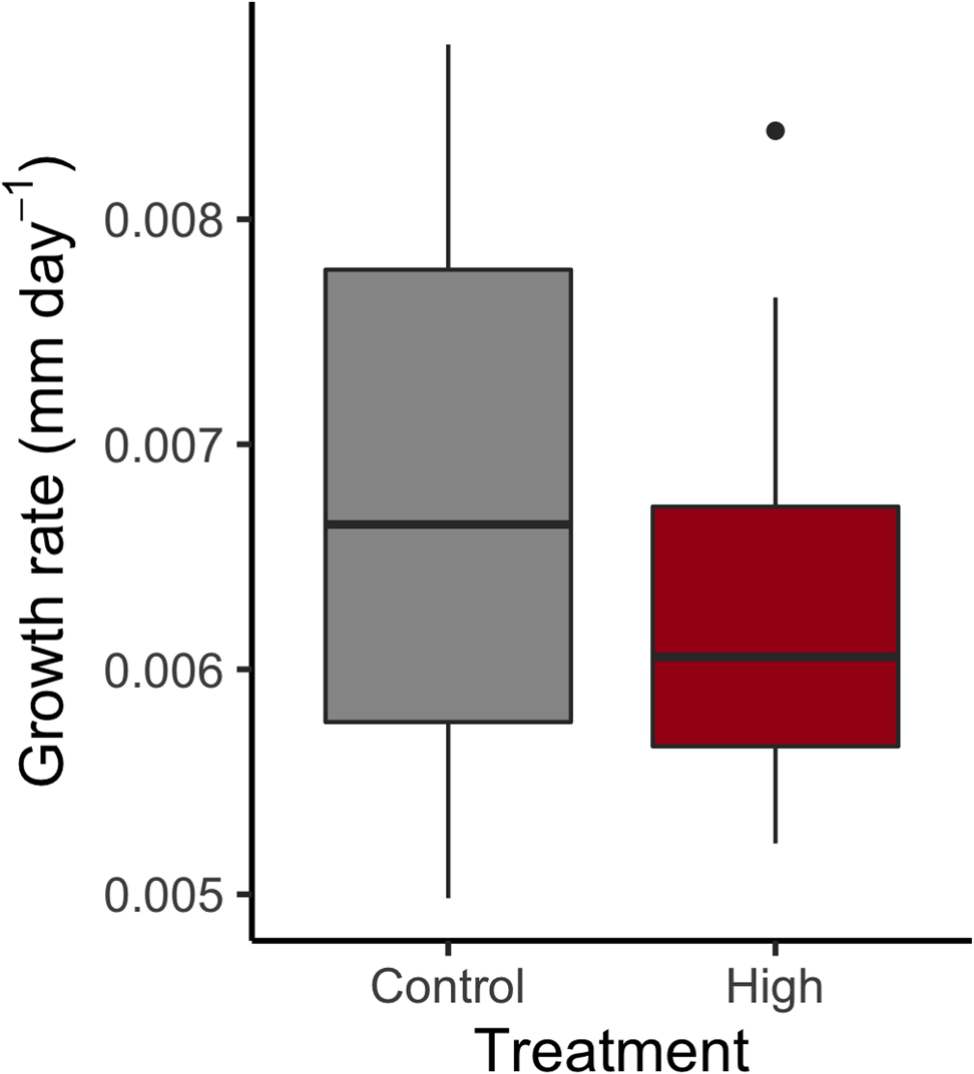
Growth rate (mm day^−1^) of *Enallagma cyathigerum* larvae in the single experiment in constant (23 °C) and temperature variation (20–26 °C) treatments. Whiskers represent minimum and maximum values excluding outliers.

Growth rate did not differ between temperature treatments (Fig. 2, F_1, 29_ = 1.58, p = 0.22). Under constant temperatures 20% (4 larvae) died and under high variation 25% (5 larvae) died throughout the experiment. No significant difference was detected for the mortality rate between the constant temperature (control) and the high temperature variation (Supplementary Fig. S2A, F_1, 38_ = 0.14, p = 0.714).

### Interaction experiment

**Figure 3:**
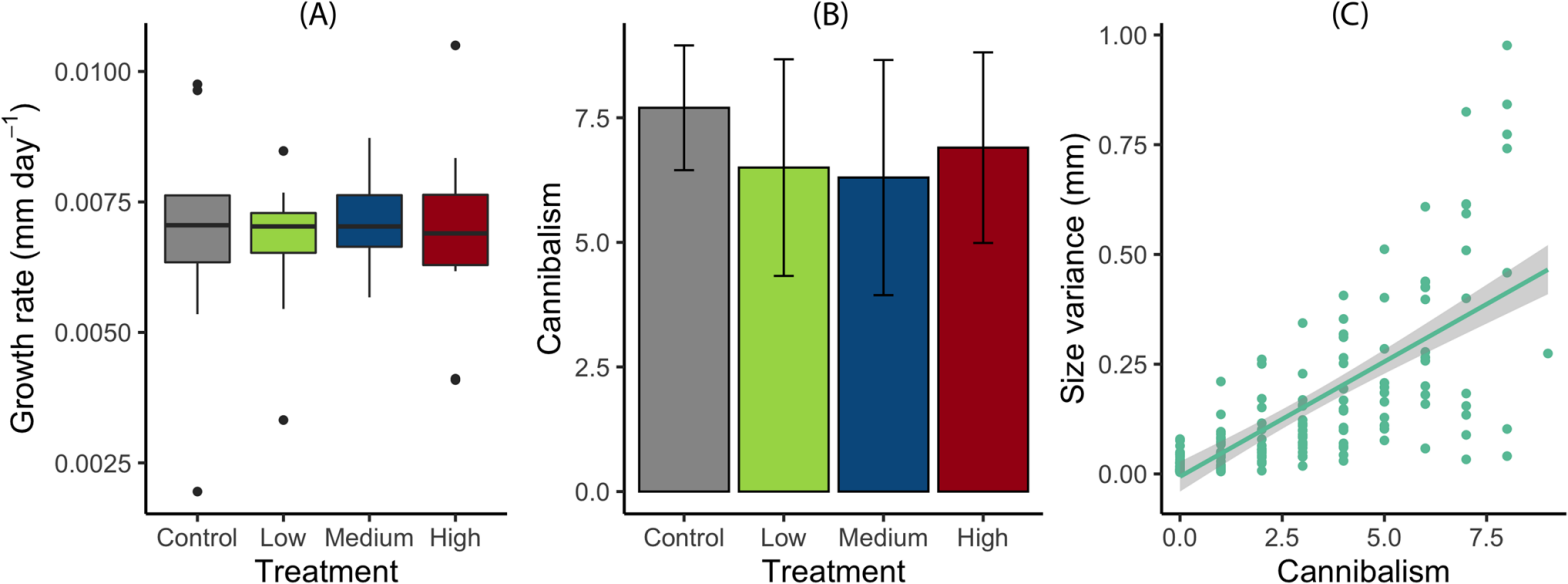
Interaction experiment. (**A**) Growth (whiskers represent minimum and maximum values excluding outliers) and (**B**) cannibalism based on the number of cannibalized larvae with an initial density of 10 individuals in constant temperature (23 °C) and across temperature variation treatments on three levels: low 22–24 °C, medium 21–25 °C, high 20–26 °C. Error bars indicate standard deviation; and (**C**) the correlation between cannibalism and size variance of *Enallagma cyathigerum* larvae.

The number of larvae decreased from an initial of 400 to 125 over time due to cannibalism (Supplementary Fig. S2C). Based on the final count of larvae, the temperature treatment did not explain much of the variation in cannibalism (Tjur’s r^2^ = 0.017), and the difference in the number of cannibalized (i.e., missing) larvae across temperature variation was not significant (Fig. 3B, Table 1). Cannibalism was significantly positively correlated with initial body size variance (p < 0.001, r^2^ = 0.42, F_1,168_ = 121.8, Fig. 3C). Growth rate did not differ significantly among temperature treatments (Fig. 3A, Table 1). The distribution of size variance was equal across temperature variation treatments (Supplementary Fig. S3A), and initial and final body sizes did not differ significantly across treatments (Supplementary Fig. S3B, C, Table 1). The temperature treatment had no significant effect on the intrinsic mortality (Table 1).

**Table 1:** Statistical results of the interaction experiment.

Cannibalism	*Df*	Deviance	Residuals	*Df* residuals	Pr(> Chi)
Temperature	3	6.8545	396	520.31	0.08

Growth	*Df*	Sum Sq	Mean Sq	F value	Pr(> F)
Temperature	3	1.25e−06	4.158e−07	0.144	0.933
Residuals	35	1.01e−04	2.886e−06		

Initial body size	*Df*	Sum Sq	Mean Sq	F value	Pr(> F)
Temperature	3	0.0069	0.0023	0.679	0.571
Residuals	36	0.1230	0.0034		

Final body size	*Df*	Sum Sq	Mean Sq	F value	Pr(> F)
Temperature	3	0.388	0.1293	0.465	0.709
Residuals	35	9.738	0.2782		

Intrinsic mortality	*Df*	Sum Sq	Mean Sq	F value	Pr(> F)
Temperature	3	0.097	0.03221	0.448	0.719
Residuals	354	25.426	0.07182		

Temperature refers to the variation treatment with three levels between 20 and 26 °C and a constant with 23 °C.

### Natural pond water temperature

The FLake model water temperature at the pond where we collected the *E. cyathigerum* larvae is given in supplementary figure S4. From June until September the minimum and maximum water temperature was 15.4 and 27.5 °C, respectively. Early in the growth season, the water temperatures varied between 15 and 20 °C (Supplementary Fig. S4), and later between 20 and 27 °C. For example, the estimated water temperature on 26 July was 26.5 °C, on 1 August 23.5 °C and on 7 August 19.8 °C (Supplementary Fig. S4). Note that: (1) some of the fluctuations are similar to the one-week temperature cycles utilized in our experiment, and that (2) an increase in water temperature of about 3 °C as predicted by climate change models of Sweden^60^, covers the temperature range we used in our experiment. For example, an increase of 3 °C in June and August would result in a mean water temperature of about 23 °C. The mean annual air temperature for Uppsala county is predicted to increase with 1.9 °C (RCP2.6), 3.1 °C (RCP4.5) and 4.8 °C (RCP8.5) for 2071–2100 compared to the reference period 1971–2000^63^.

## Discussion

Understanding the impact of naturally occurring temperature fluctuations is essential for making predictions on biodiversity changes in the future. While past studies have focused on how temperature variation might affect survival, growth, and development, few have focused on how temperature variation affects competitive interactions. Results from this study showed that temperature variations at three different levels of amplitude, but all with the same mean temperature, did not affect mortality, growth, or cannibalism in larvae of the damselfly *Enallagma cyathigerum*. Since our study reflected natural temperature conditions, we suggest that the increase in temperature variation predicted by climate models will not affect growth and cannibalism in the study system compared to the changes predicted with increases to mean temperatures. We propose that the absence of a difference between temperature variation and the control treatment was because the temperature variations were within the approximately linear part to the left of the thermal optimum of the TPC. Nevertheless, this part of the TPC is within the range of temperatures during the main growth season of the damselfly larvae.

We found that cannibalism occurred in intraspecific treatments, however, it was not significantly affected by temperature variations reflecting recent and future temperature conditions at the sample location. With increasing temperatures, metabolic rates typically increase exponentially^67,68^, leading to higher activity in organisms^69^. In line with this, one would expect higher encounter rates at higher temperatures, particularly in a container where dispersal of individuals is limited, and thus, higher rates of cannibalism can occur. Higher predation at higher temperatures has been observed for predator–prey systems in several studies^56,70,71^. To our knowledge, no previous research has been published on the effect of temperature variation on cannibalism. Temperature variation studies combine periods of higher and lower temperatures over a specific time scale, but the biotic response is not necessarily neutral due to the asymmetric shape of thermal performance curves, which makes predictions generally difficult. In our study, higher and lower activity during warm and cold periods might lead to an overall neutral response because the temperature variations are within the approximately linear part to the left of the thermal optimum of the TPC. In addition, no effect of cannibalism or growth rate was detected in the interaction experiment. Under the assumption that individual growth rate was not affected by temperature variation directly (see results from the single larvae experiment), the absence of a growth rate effect is not surprising given the non-significant cannibalism effect. Had we observed a significant cannibalism effect, we would have expected a difference in growth rate, higher in treatments with higher cannibalism.

There was a significant correlation between initial larval size variance and cannibalism. This can be referred to as size-dependent cannibalism^38^, where cannibalism only occurs if the victim to cannibal body size ratio exceeds a critical value^72–74^. This typical correlation, however, is very unlikely to have confounded our results as the size variance was equally distributed across temperature treatments. Counting the number of missing larvae in our experiments was considered as a quantitative proxy for cannibalism. Since the larvae were counted on a weekly basis, there was not enough time for carcasses to be decomposed during this time and damselfly larvae are not known to feed on carcasses. Thus, larvae preying on other living larvae is the only reasonable explanation for the missing larvae. Moreover, cannibalism was observed visually several times throughout the experiment.

Growth rates did not differ significantly between constant and fluctuating temperatures in the single larvae experiment, an outcome contrasting with our hypothesis. A decrease in performance (i.e., growth) as soon as the temperature falls outside the thermal optimum of the species is expected. In our experiment it turned out that the temperature variation we used were on the left side of the thermal optimum. Despite the overall non-linearity of the relationship between growth and temperature on this side of the TPC, we suggest that the change along the slope of the TPC causes a very small difference below and above the mean temperature used (23 °C), and hence, the temperature fluctuation effect is canceled out. In fact, when we calculated the difference of the thermal performance slopes between 20–23 °C and 23–26 °C (the warm and cold temperature ranges used in our fluctuation cycle with a constant of 23 °C), the difference between the slopes is very small but slightly flatter above the mean (Supplementary Discussion S1). In line with our hypothesis, this translates into an overall very small decrease in performance comparing the constant control with fluctuating temperatures: a trend which was indeed observed for growth, as well as cannibalism.

Other studies on growth rate at fluctuating and constant temperatures have found conflicting results. For instance, in an experimental study on the fruit fly *Drosophila melanogaster* growth rates increased with variations at low temperatures but decreased with variations around the thermal optimum in comparison to constant control temperatures^28^. In another study, daily temperature fluctuations reduced growth rates in the damselfly *Ischnura elegans*; however, the effects of variations were also strongly related on mean temperatures and only present in higher temperatures^75^. The same pattern was demonstrated in an experiment on tobacco hornworms, *Manduca sexta*, where diurnal temperature variations impacted growth but depending on the mean temperature^24^. Hence, not surprisingly, the outcome highly depends on the mean temperature and the thermal optimum. Our study and previous findings support our assumption that the link to the TPC is highly important for interpreting results of thermal variation studies.

Increases in temperature under projected climate change scenarios are likely to impact the physiology and survival of damselfly larvae^65,76^. The maximum temperature used in our study was 28 °C, under which growth rates flattened, but did not decrease yet. The thermal optimum we estimated using the O’Neill model matches with that found in other Coenagrionidae damselfly species. In a study on *E. cyathigerum*^52^, growth started to decrease above ~ 29 °C. Similar patterns with decreasing growth rates under increasing temperature conditions have been shown in several related *Enallagma* and other Odonata species^65,76,77^. This supports the decrease in growth rates after the thermal optimum of the TPC as estimated from the O’Neill model, i.e. 26.9 °C. Water temperatures higher than 30 °C are likely under projected climate change scenarios for the study region Uppsala, with a predicted increase of average air temperatures of more than 3 °C under RCP4.5^63^. Lake surface temperatures are expected to follow and even exceed the increases in air temperature, as empirically shown in several European lakes^78^. Some aquatic organisms might be able to shift their distribution to colder layers. However, most damselfly larvae are categorized as climbers spending more time on macrophytes rather than moving around in open water^79,80^.

In general, the intrinsic mortality in the single larvae experiment and in the interaction experiment (excluding cannibalism) was low, implying that the temperature range used in this experiment (20–26 °C) did not have lethal effects and are within the species thermal breadth. Furthermore, intrinsic mortality rates did not differ across temperature variation treatments, which suggests that the higher temperature variations used in this study did not affect the survival probability.

Predicting the effects of global climate change on species interactions is one of the most pressing challenges for ecologists. Ectotherms are particularly sensitive towards thermal conditions and show severe declines in abundances in respond to climate change^1,20,81^. Whilst the major body of literature focused on rising mean temperatures to date, many studies neglected the increasing frequency of temperature variations and its intricated consequences on biotic interactions. Our work contributes to this gap in our knowledge and shows, that temperature fluctuations do not necessarily cause stronger or weaker effects than more even temperatures. Weekly temperature variations within a recent natural range did not significantly affect growth, mortality, or cannibalism in *Enallagma cyathigerum* larvae, presumably due to the small changes in the slope along the TPC within the experimental temperature range. One can expect to receive this result also in other studies and systems, when the temperature variation interval is to the left of the species thermal optimum. We thus highlight the importance of thermal performance curves in thermal variation studies. Besides these main findings, a clear correlation between body size variance and cannibalism was found, supporting previous evidence of size-dependent cannibalism.

## Supplementary Information

Supplementary Information.

## Data Availability

The datasets generated and analysed during the current study are available in the Github repository, https://github.com/KLHolzmann/ThermalVariation.
