# Supplementary material for: Weak effects on growth and cannibalism under fluctuating temperatures in damselfly larvae
Source: Sci Rep. 2022 Jul 28;12:12910. doi: 10.1038/s41598-022-17192-1 (PMC9334275; doi:10.1038/s41598-022-17192-1)
Supplement: Supplementary file 1 — Supplementary Information. [file 41598_2022_17192_MOESM1_ESM.pdf]

Supplementary Material for

Weak effects on growth and cannibalism under fluctuating temperatures in damselfly larvae

Kim Lea Holzmann[1,*], Chloé Charrier[1], and Frank Johansson[1]

[1]Uppsala University, Department of Ecology and Genetics, Uppsala, 75236, Sweden

* kim.holzmann@evobio.eu

Supplementary Figures

Experimental Set-up

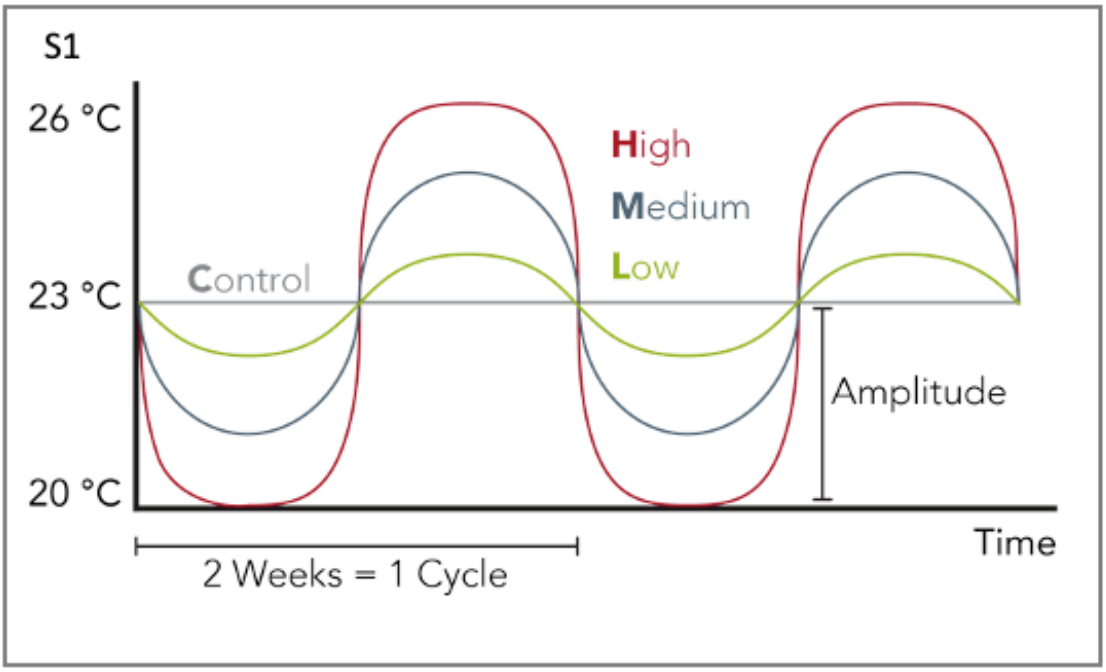

Fig. S1: Experimental temperature fluctuations ranging from 20–26°C on three levels, all with an average of 23°C, same as the constant control treatment. The interaction experiment run for 4 cycles, the single experiment on control and high variation level for 3 cycles.

Mortality

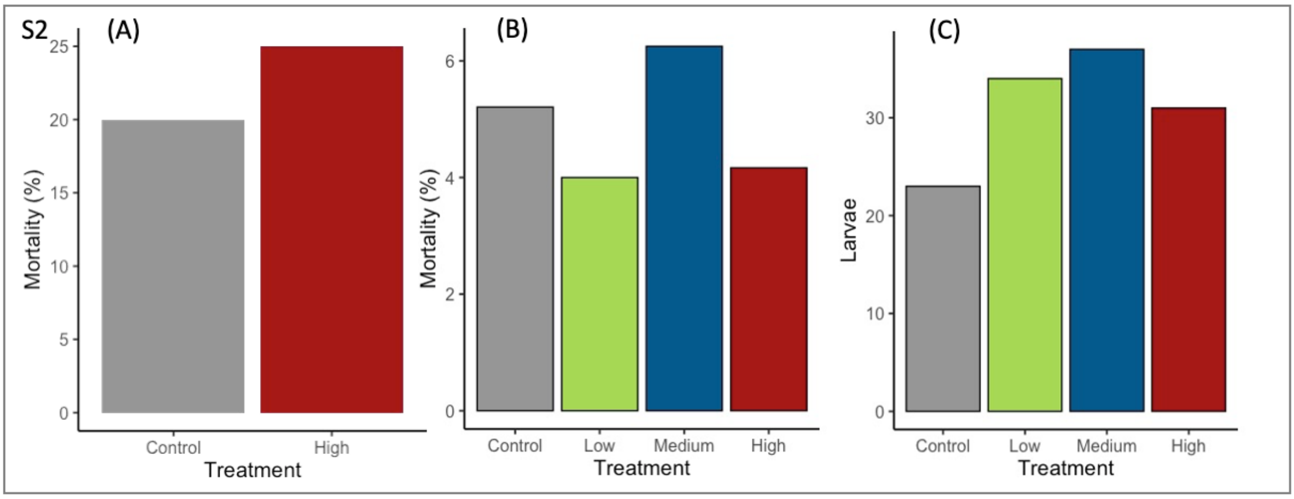

Fig. S2: Mortality of Enallagma cyathigerum larvae in (A) the single experiment under a constant temperature (23°C) and high temperature variation (20–26°C), and (B&C) the interaction experiment in a constant temperature (23°C) and across temperature variation treatments on three levels: low 22–24°C, medium 21–25°C, high 20–26°C; with (B) intrinsic mortality excluding death due to cannibalism and (C) the number of alive larvae (excluding carcasses) at the end of the experiment.

Body Sizes

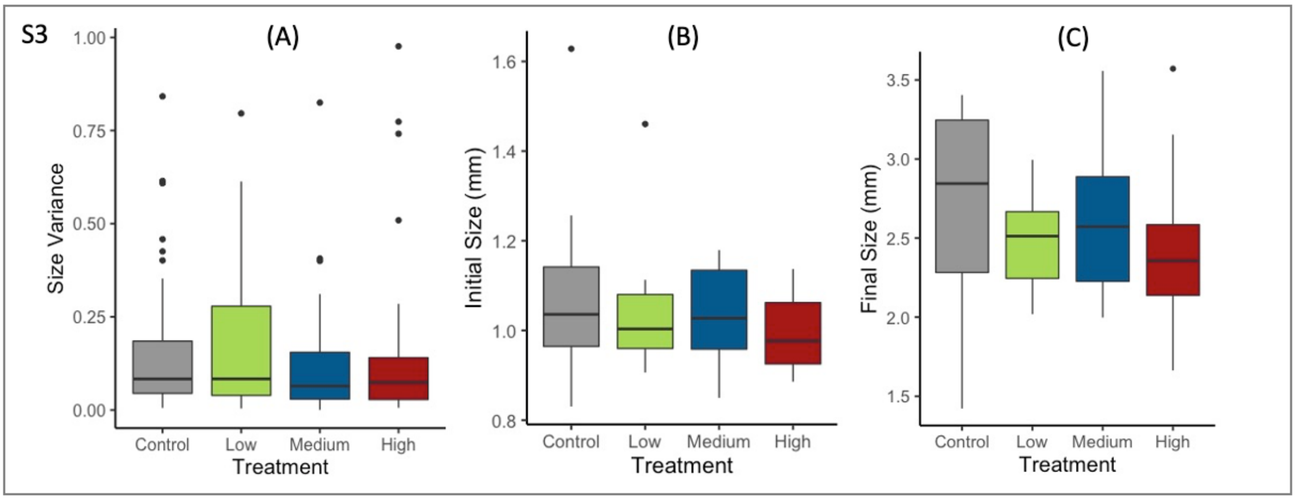

Fig. S3: Body sizes (head width in mm) of Enallagma cyathigerum larvae in the interaction experiment in a constant temperature (23°C) and across temperature variation treatments on three levels: low 22–24°C, medium 21–25°C, high 20–26°C, with (A) all treatments having similar values of body size variance (i.e., no confounding effect), (B) initial body sizes and (C) final body sizes at the end of the experiment. Whiskers represent minimum and maximum values excluding outliers.

Lake Temperatures

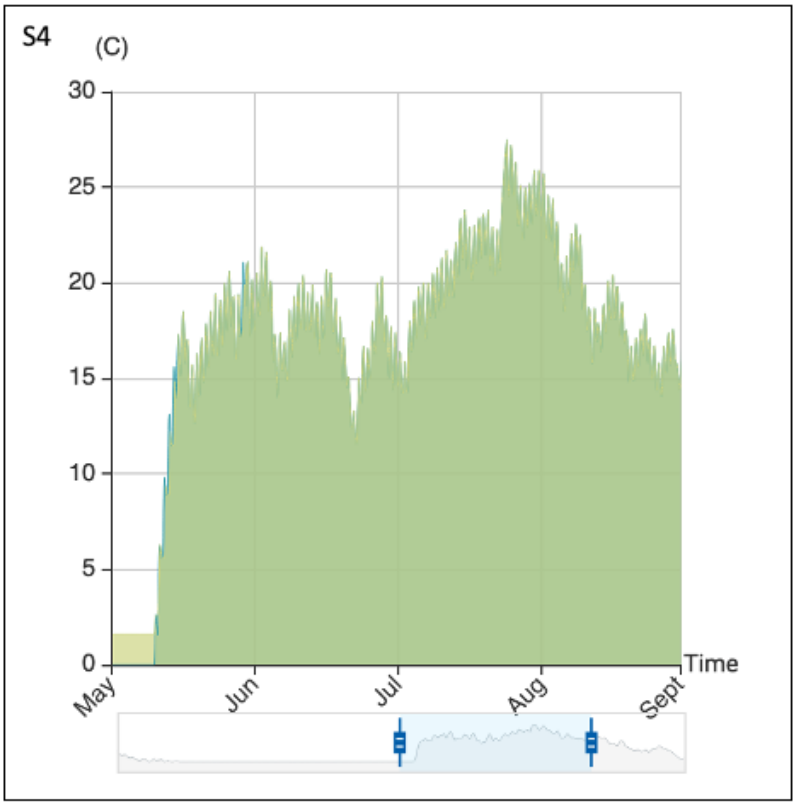

Fig. S4: Result of the lake model FLake to estimate natural temperature in the pond where E. cyathigerum larvae were collected, showing the water during the main growth season of damselfly larvae, May to September temperature (surface and bottom temperature overlap due to the shallow depth of the pond). Note the approximately weekly fluctuation patterns particularly in late season.

Supplementary Discussion S1

Following the assumption of linearity, we calculated the difference of the thermal performance slopes between 20–23°C (m_1) and 23–26° (m_2), the warm and cold temperature ranges used in our fluctuation cycle with a constant of 23°C.

$$m_1 = \frac{rate_2 - rate_1}{temperature_2 - temperature_1} = \frac{0.059\, mm * day^{-1} - 0.046\, mm * day^{-1}}{23°C - 20°C} = 0.0043$$

$$m_2 = \frac{0.068\, mm * day^{-1} - 0.059\, mm * day^{-1}}{26°C - 23°C} = 0.003$$

$$m_1 - m_2 = 0.0013$$

The difference between the slopes is very small, which means a decrease of performance during the colder period has a very similar effect size than an increase of performance during the warmer period. Thus, over a full cycle, these effects would mostly cancel each other out.
